# CPNE1 promotes non-small cell lung cancer progression by interacting with RACK1 via the MET signaling pathway

**DOI:** 10.1186/s12964-021-00818-8

**Published:** 2022-01-31

**Authors:** Anqi Wang, Wen Yang, Yue Li, Yang Zhang, Jieqi Zhou, Ruochen Zhang, Weijie Zhang, Jianjie Zhu, Yuanyuan Zeng, Zeyi Liu, Jian-an Huang

**Affiliations:** 1 Department of Pulmonary and Critical Care Medicine, The First Affiliated Hospital of Soochow University, Suzhou, 215006 China; 2 Institute of Respiratory Diseases, Soochow University, Suzhou, 215006 China; 3 Suzhou Key Laboratory for Respiratory Diseases, Suzhou, 215006 China

**Keywords:** Non-small cell lung cancer (NSCLC), Copine 1 (CPNE1), Receptor for activated C kinase 1 (RACK1), Mesenchymal-epithelial transition tyrosine kinase receptor (MET), Epidermal growth factor receptor (EGFR)

## Abstract

**Background:**

Non-small cell lung cancer (NSCLC) is the most common type of lung cancer and the most lethal tumour worldwide. Copine 1 (CPNE1) was identified as a novel oncogene in NSCLC in our previous study. However, its specific function and relative mechanisms remain poorly understood.

**Methods:**

The biological role of CPNE1 and RACK1 in NSCLC was investigated using gene expression knockdown and overexpression, cell proliferation assays, clonogenic assays, and Transwell assays. The expression levels of CPNE1, RACK1 and other proteins were determined by western blot analysis. The relationship between CPNE1 and RACK1 was predicted and investigated by mass spectrometry analysis, immunofluorescence staining, and coimmunoprecipitation. NSCLC cells were treated with a combination of a MET inhibitor and gefitinib in vitro and in vivo.

**Results:**

We found that CPNE1 facilitates tumorigenesis in NSCLC by interacting with RACK1, which further induces activation of MET signaling. CPNE1 overexpression promoted cell proliferation, migration, invasion and MET signaling in NSCLC cells, whereas CPNE1 knockdown produced the opposite effects. In addition, the suppression of the enhancing effect of CPNE1 overexpression on tumorigenesis and MET signaling by knockdown of RACK1 was verified. Moreover, compared to single-agent treatment, dual blockade of MET and EGFR resulted in enhanced reductions in the tumour volume and downstream signaling in vivo*.*

**Conclusions:**

Our findings show that CPNE1 promotes tumorigenesis by interacting with RACK1 and activating MET signaling. The combination of a MET inhibitor with an EGFR-TKI attenuated tumour growth more significantly than either single-drug treatment. These findings may provide new insights into the biological function of CPNE1 and the development of novel therapeutic strategies for NSCLC.

**Video Abstract**

**Supplementary Information:**

The online version contains supplementary material available at 10.1186/s12964-021-00818-8.

## Background

Lung cancer, among which non-small cell lung cancer (NSCLC) accounts for more than 80% of cases, has long been the leading cause of cancer-related mortality worldwide [1, 2]. Although different types of new treatments have been developed, the 5-year overall survival rate remains less than 20% because the pathogenic mechanism and developmental process of NSCLC are complex and still poorly understood [3]. Thus, we believe it is of great interest to continue exploring the pathogenesis of NSCLC and develop new therapeutic strategies to improve NSCLC treatment.

In our previous study, we found Copine 1 (CPNE1) to be an important oncogene in NSCLC. It is directly targeted by miR-335-5p and promotes cell proliferation and motility in NSCLC [4–6]. CPNE1 includes two tandem C2 domains at the N-terminus and an A domain at the C-terminus. The C2 domains act as calcium-dependent phospholipid-binding motifs and may be involved in cell signaling and membrane trafficking pathways [7, 8]. The A domain is named after von Willebrand factor, a plasma and extracellular matrix protein. The A domain has been studied in integrins and several extracellular matrix proteins and appears to function as a protein-binding domain [9]. Though we discovered the expression and function of CPNE1 in NSCLC, few studies have focused on the exact intracellular signaling mechanism. Here, we seek to explore the molecular mechanism by which CPNE1 promotes NSCLC tumorigenesis and executes the CPNE1-centred regulatory network in NSCLC.

We then performed proteomic analysis to investigate whether CPNE1 interacts with other proteins to activate downstream signaling pathways and found that receptor for activated C kinase 1 (RACK1) was most likely related to CPNE1. RACK1 is a member of the tryptophan-aspartate repeat (WD-repeat) family of proteins, also known as a highly conserved intracellular adaptor protein. It has seven β-propeller blades that serve as binding sites for multiple interaction partners and hence possess significant homology to the β subunit of G-proteins (Gβ) [10]. RACK1 can also act as a scaffolding protein, making it a key mediator of various pathways that contribute to numerous aspects of cellular function [11]. Studies have identified that RACK1 plays an important role in different types of human cancers, such as breast cancer [12], hepatocellular carcinoma [13], melanoma [14], and lung adenocarcinoma [15, 16]. As RACK1 is a crucial factor in tumour progression and development, we sought to investigate its role in NSCLC and its relation with CPNE1, as well as the underlying mechanism.

Next, the mesenchymal-epithelial transition tyrosine kinase receptor (MET or c-MET) pathway was detected as a potential regulatory pathway activated by the CPNE1/RACK1 complex. MET is a receptor tyrosine kinase (RTK) activated by its ligand, hepatocyte growth factor (HGF), and mediates several downstream signaling pathways, including the MEK/ERK, PI3K/AKT, and JAK/STAT pathways [17]. Under physiological conditions, the MET pathway can take part in embryogenesis, wound healing and tissue regeneration [18]. Genomic MET alterations, including mutations and gene amplification, can cause aberrant activation of the MET signaling pathway, promoting tumour cell growth, survival, migration, and invasion in a variety of tumours. It is worth noting that both MET mutations and amplification are often detected in NSCLC and are associated with poor prognosis and resistance to epidermal growth factor receptor tyrosine kinase inhibitors (EGFR-TKIs) [19–21]. While a high level of MET amplification is recognized by the NCCN guidelines to influence treatment decisions for NSCLC, MET inhibitors like capmatinib, tepotinib, and savolitinib have come into our sight, the development of acquired resistance to these new agents remain a problem, hence more clinical trials are needed to confirm the efficacy of MET inhibitors or other combined treatments [22–24]. Meanwhile, our previous work showed that CPNE1 can activate the EGFR signaling pathway [4], and a bispecific antibody targeting EGFR and MET called amivantamab has been discovered recently [25]. Hereby, we intend to broaden the use of MET inhibitors in CPNE1-overexpressing patients and explore a new scheme of combined treatment with MET inhibitors and EGFR inhibitors.

In this study, we demonstrated that CPNE1 can promote the malignant phenotype of NSCLC by interacting with RACK1 and subsequently activating the MET signaling pathway. We also found that combined targeting of MET and EGFR can result in enhanced inhibition of tumorigenesis both in vitro and in vivo, indicating a new therapeutic strategy for treating NSCLC.

## Methods

### Cell lines and cell culture

The NSCLC cell lines A549 (wild-type EGFR/exon2 G12S KRAS) and HCC827 (exon21 L858R EGFR/wild-type KRAS) (lung adenocarcinoma cell lines) were purchased from the Cell Bank of the Chinese Academy of Sciences (Shanghai, China). Cells were incubated at 37 °C in a humidified air atmosphere containing 5% carbon dioxide in RPMI 1640 medium with 10% foetal bovine serum (FBS), 100 μg/ml penicillin (Sigma-Aldrich, USA), and 100 μg/ml streptomycin (Sigma-Aldrich, USA). All cell lines were mycoplasma-free and authenticated by quality examinations of their morphology and growth profiles.

### Clinical NSCLC tissue samples

Fourteen paired patient samples of NSCLC tissues and matched adjacent noncancerous tissues were obtained from the First Affiliated Hospital of Soochow University between 2017 and 2020. The patients had been diagnosed with NSCLC based on their histological and pathological characteristics according to the Revised International System for Staging Lung Cancer. Tissue samples were acquired during therapeutic surgery of patients who had not previously received any antitumour treatment. Upon resection, human surgical specimens were immediately frozen in liquid nitrogen and stored at − 80 °C. Informed consent was obtained from all patients, and the research was approved by the Ethics Committee of Soochow University.

### RNA interference

Predesigned short interfering RNA (siRNA) sequences that target different coding regions of CPNE1 or RACK1 were directly synthesized by GenePharma (Suzhou, China). The target sequences are listed in Additional file 1: Table S1. Scrambled siRNA was used as a negative control. Cells were transiently transfected with 100 pmol of siRNA sequences using Lipofectamine 2000 (Invitrogen, USA). After 72 h of transfection, the cells were harvested for further experiments.

### RNA isolation and quantitative real-time PCR assays

RNA isolation, cDNA synthesis and real-time quantitative reverse transcription PCR (qRT-PCR) was performed as previously described [26]. The specific primers for target genes are listed in Additional file 2: Table S2. Gene expression levels were quantified according to the comparative ΔΔCt method, and β-actin was used as the internal control.

### CPNE1-overexpressing plasmid construction and transfection

The CPNE1-overexpressing plasmid was constructed as described in our previous work [4]. The empty vector served as a negative control. Human embryonic kidney 293T cells were cultured in Dulbecco’s modified Eagle’s medium (DMEM) containing 10% FBS at 37 °C in a humidified 5% CO_2_ incubator for 48 h. After incubation, the packaged lentiviruses were collected and used to infect A549 and HCC827 cells. After 2 days, stable cells were selected with 400 μg/ml G418 (Amresco, Solon, OH, USA).

### Cell proliferation assay

Cells were seeded in 96-well plates at a density of 2 × 10^3^ cells per well and further grown under normal culture conditions for 24, 48 and 72 h. Cell viability was determined using Cell Counting Kit-8 (Boster, Wuhan, China) according to the manufacturer’s instructions. For clonogenic assay, cells were diluted in complete culture medium, and 300 cells were reseeded in a 60-mm plate. After incubation for 7–14 days, depending on the cell growth rate, foci formed by at least 50 cells were stained with 0.1% crystal violet and counted. The experiment was performed in triplicate.

### Cell migration and invasion assays

Cell migration and invasion assays were performed in a 24-well plate with 8 μm pore size chamber inserts (Corning, USA). A total of 5 × 10^4^ cells (migration assays) and 1 × 10^5^ cells (invasion assays) were inoculated into the upper chambers. For the invasion assays, the wells contained membranes coated with Matrigel (Corning, USA), which was diluted with serum-free culture medium. In both assays, cells were suspended in 200 μl of 1640 without FBS. In the lower chamber, 800 μl of 1640 supplemented with 10% FBS was added. After 24 h, the cells that remained in the upper chamber were removed with a cotton swab, and the cells that moved to the bottom surface of the membrane were fixed with 100% methanol and stained with 0.1% crystal violet for 20 min. Then, the cells were imaged and counted under a microscope. Assays were conducted independently three times.

### Western blot analysis

Western blot analysis was performed as previously described [26]. The primary antibodies used in this study were anti-CPNE1 (ab155675) (Abcam, UK), anti-RACK1 (sc-17754) (Santa Cruz, USA), anti-pEGFR (Tyr1068) (1H12) (#3777), anti-EGFR (#4267), anti-pAKT (Ser473) (D9E) (#4060), anti-AKT (#4685), anti-pERK (Thr202/Tyr202) (D13.14.4E) (#4370), anti-ERK (#4695), and anti-β-actin (#3700) (Cell Signaling Technology, USA). The bands were developed by an electrochemiluminescence reagent, imaged with a ChemiDoc XRS + (Bio-Rad, USA), and finally quantified with ImageJ software (National Institutes of Health, Bethesda, MD, USA).

### Co-immunoprecipitation

Co-immunoprecipitation was performed with A549 and HCC827 cells. Equal amounts of protein (3000 μg) were preprocessed by protein A/G magnetic beads (Thermo Scientific, USA). After 2 h, the lysates were incubated with antibodies at 4 °C overnight, followed by overnight incubation with beads. The next day, the beads were gently washed five times with phosphate-buffered saline (PBS) containing 1% Triton X-100, and the beads were then incubated with 2 × protein loading buffer at 100 °C for 10 min. IgG-bound, CPNE1-bound, or RACK1-bound proteins were separated using SDS-PAGE and subjected to western blot analysis.

### Immunofluorescence staining

Cells were seeded in 12-well plates containing pre-inserted glass slides. Then, the cells were washed with PBS 24 h later at a confluence of 40–50%. Cells were fixed with 4% paraformaldehyde for 30 min afterwards, followed by permeabilization with 0.5% Triton X-100 solution for an additional 20 min. Next, 5% bovine serum albumin was added to function as the blocking buffer. The primary antibodies, anti-CPNE1 (Abcam, UK) and anti-RACK1 (Santa Cruz, USA), and corresponding secondary antibodies conjugated to Cy3 and FITC were used successively.

### Mass spectrometry analysis

The cell lysates from H1299 cells transfected with human pcDH-Flag-CPNE1 plasmid and control vector were lysed with lysis buffer mixed with proteinase cocktail inhibitor (Roche, Branford, CT, USA). The protein profile was analysed using a human Phospho-RTK Array Kit, ARY001B (R&D Inc. Minneapolis, MN, USA) according to the manufacturer’s protocols (Additional file 3: Table S3). The affinity-purified samples were analysed with an Orbitrap Elite hybrid mass spectrometer (Thermo Fisher) performed by Luming Biotechnology (Shanghai, China). Proteins with an expression ratio ≥ 1.5 and *p* < 0.05 were regarded as aberrantly regulated proteins and were clustered with the R package heatmap2. The predicted interaction among selected proteins was analysed with the network analysis tool Cytoscape.

### Animal experiments

Female BALB/c athymic nude mice (4–6 weeks old and weighing 16–20 g) were purchased from the Experimental Animal Center of Soochow University and bred under pathogen-free conditions. A549 cells overexpressing CPNE1 were suspended in 100 ml of RPMI 1640 medium and inoculated subcutaneously into the flanks of nude mice, which were randomly divided into four groups (3 mice in each group). The tumour volumes (*V*) was determined by measuring the tumour length (*L*) and width (*W*) with a Vernier calliper and applying the following formula: *V* = (*L* × *W*^2^) × 0.5. When the tumour volume reached 100–150 mm^3^, DMSO, gefitinib, JNJ-38877605, and the combination treatment were given by gavage at 5 mg/kg/d (gefitinib) and 50 mg/kg/d (JNJ-38877605) until the mice were sacrificed. All animal experiments were carried out in accordance with the Guide for the Care and Use of Experimental Animals Center of Soochow University.

### Statistical analysis

The quantitative variables are presented as the mean and S.E.M. values, and differences between the two groups were analysed by Student’s *t* test (two-tailed; *p* < 0.05 was considered statistically significant). Two-way ANOVA was used to determine the difference in cell growth between four groups for in vivo experiments. The overall survival time was defined as the length of time between surgery and death. Graphs were generated with GraphPad Prism 7.

## Results

### The role of CPNE1 in mediating NSCLC cell proliferation, migration and invasion

**Fig. 1:**
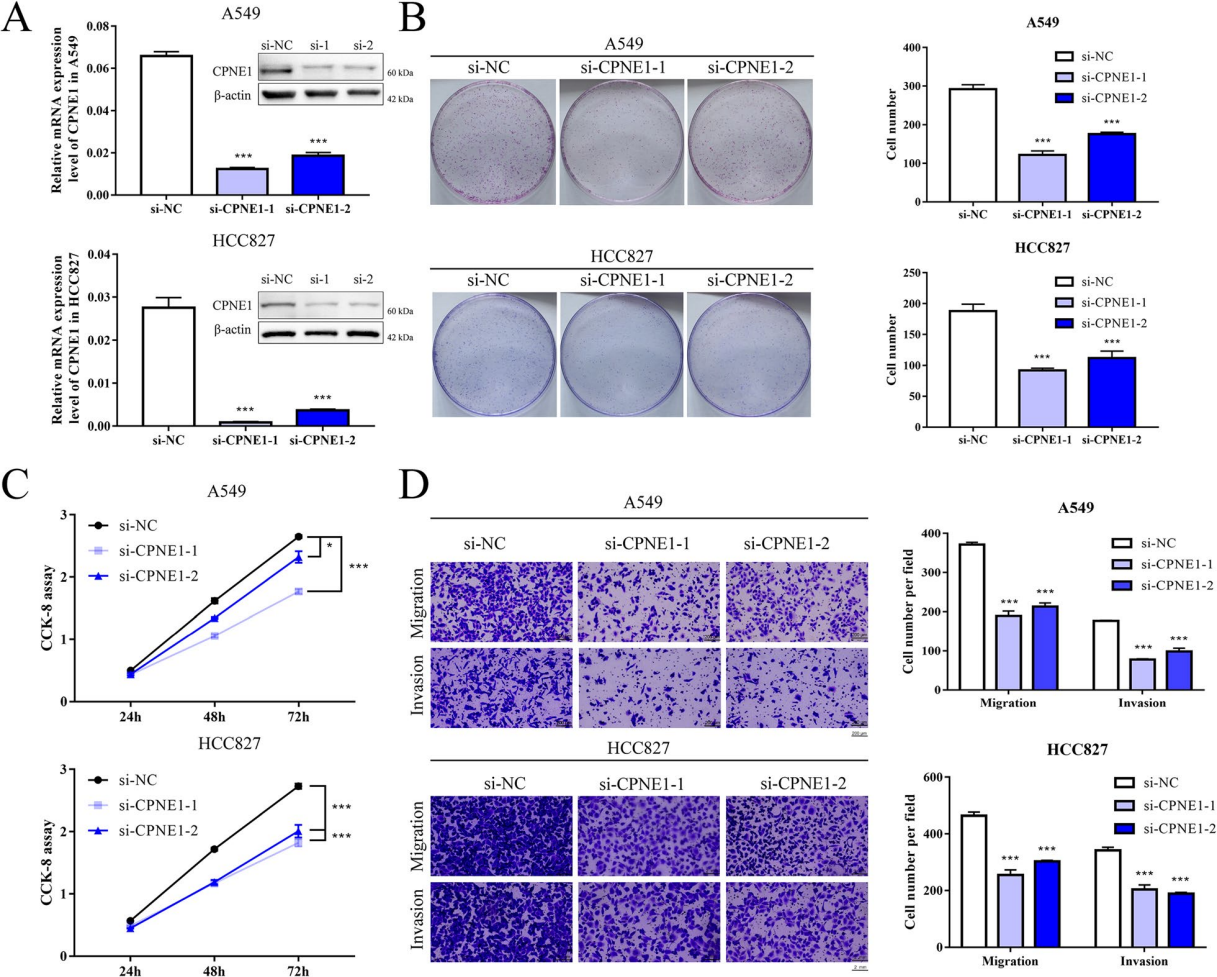
Knockdown of CPNE1 inhibited the proliferation and motility of A549 and HCC827 cells. **A** The mRNA and protein level of CPNE1 was downregulated in CPNE1 knockdown cells. **B** The clonogenic assays were performed to assess cell proliferation. **C** CCK-8 assays were also performed to analyse cell proliferation. **D** Transwell assays were performed to analyse the cell motility abilities. Data represent the mean (± SD) of three independent experiments, and unpaired *t* test was used to verify the statistical significance. *P < 0.05; **P < 0.01; ***P < 0.001

**Fig. 2:**
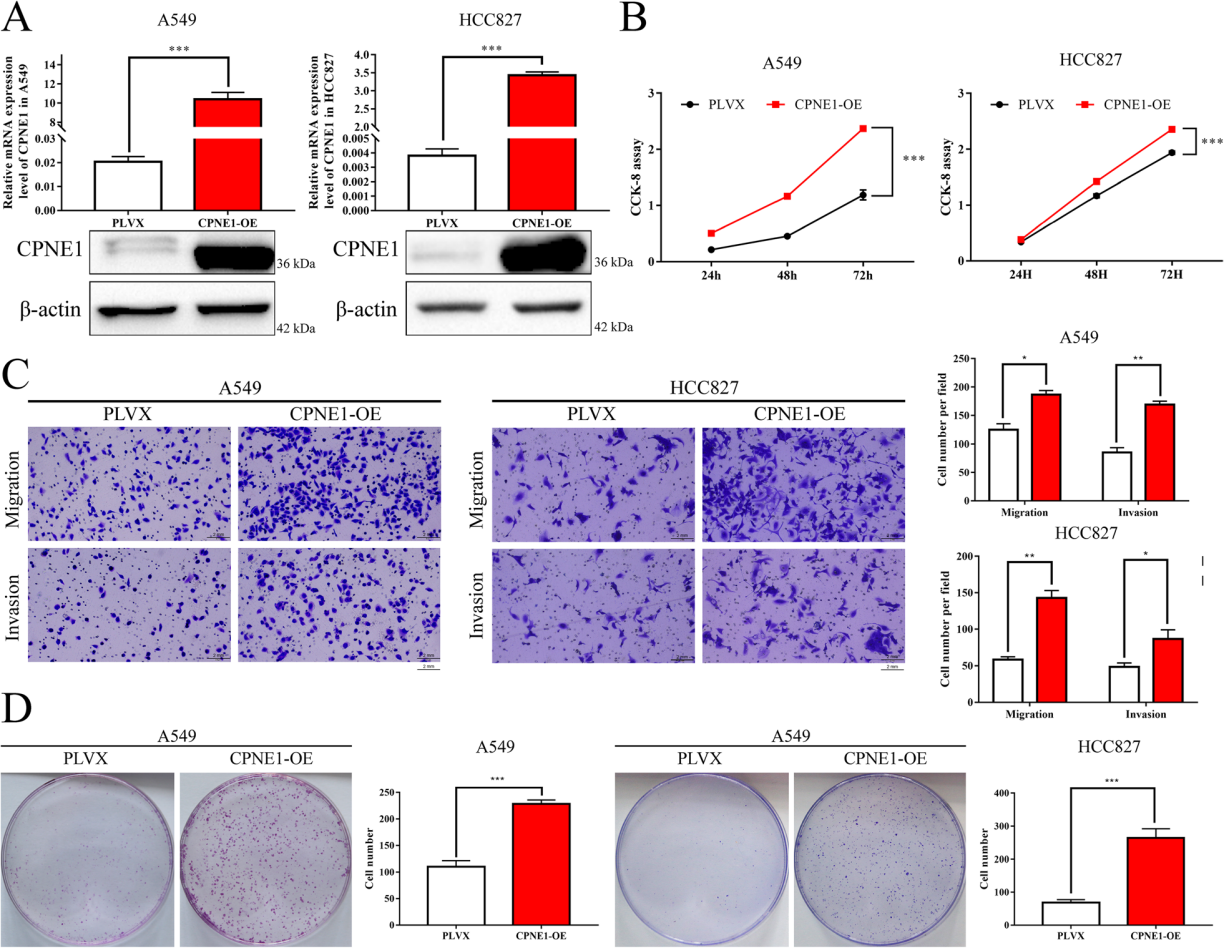
Upregulation of CPNE1 increased the proliferation and motility of A549 and HCC827 cells. **A** The mRNA and protein level of CPNE1 was increased in CPNE1-overexpressing cells. **B** CCK-8 assays were performed to analyse cell proliferation. **C** Transwell assays were performed to analyse the cell motility abilities. **D** Clonogenic assays were performed to assess cell proliferation. Data represent the mean (± SD) of three independent experiments, and unpaired *t* test was used to verify the statistical significance. *P < 0.05; **P < 0.01; ***P < 0.001

Since our previous study and data from GEPIA database (http://gepia.cancer-pku.cn/) both found that CPNE1 is upregulated in NSCLC and associated with poor prognosis [4, 5] (Additional file 4: Fig S1), we further validated the results of altered expression of CPNE1. The expression level of CPNE1 was significantly reduced after transfection with two siRNAs against CPNE1 (Fig. 1A). CCK-8 assays and clonogenic assays were performed to confirm that cell growth was significantly inhibited in CPNE1 knockdown cells compared with control cells after transfection (Fig. 1B, C). Transwell assays further demonstrated that loss of CPNE1 considerably suppressed the migration and invasion abilities of NSCLC cells (Fig. 1D). Moreover, we established A549 and HCC827 cell lines with stable overexpression of CPNE1 (Fig. 2A). The results showed that cell proliferation, migration and invasion were significantly promoted in cells overexpressing CPNE1 (Fig. 2B–D). Collectively, these data strongly suggest that CPNE1 is a crucial oncogene promoting NSCLC progression.

### CPNE1 regulates the MET signaling pathway in NSCLC cell lines

**Fig. 3:**
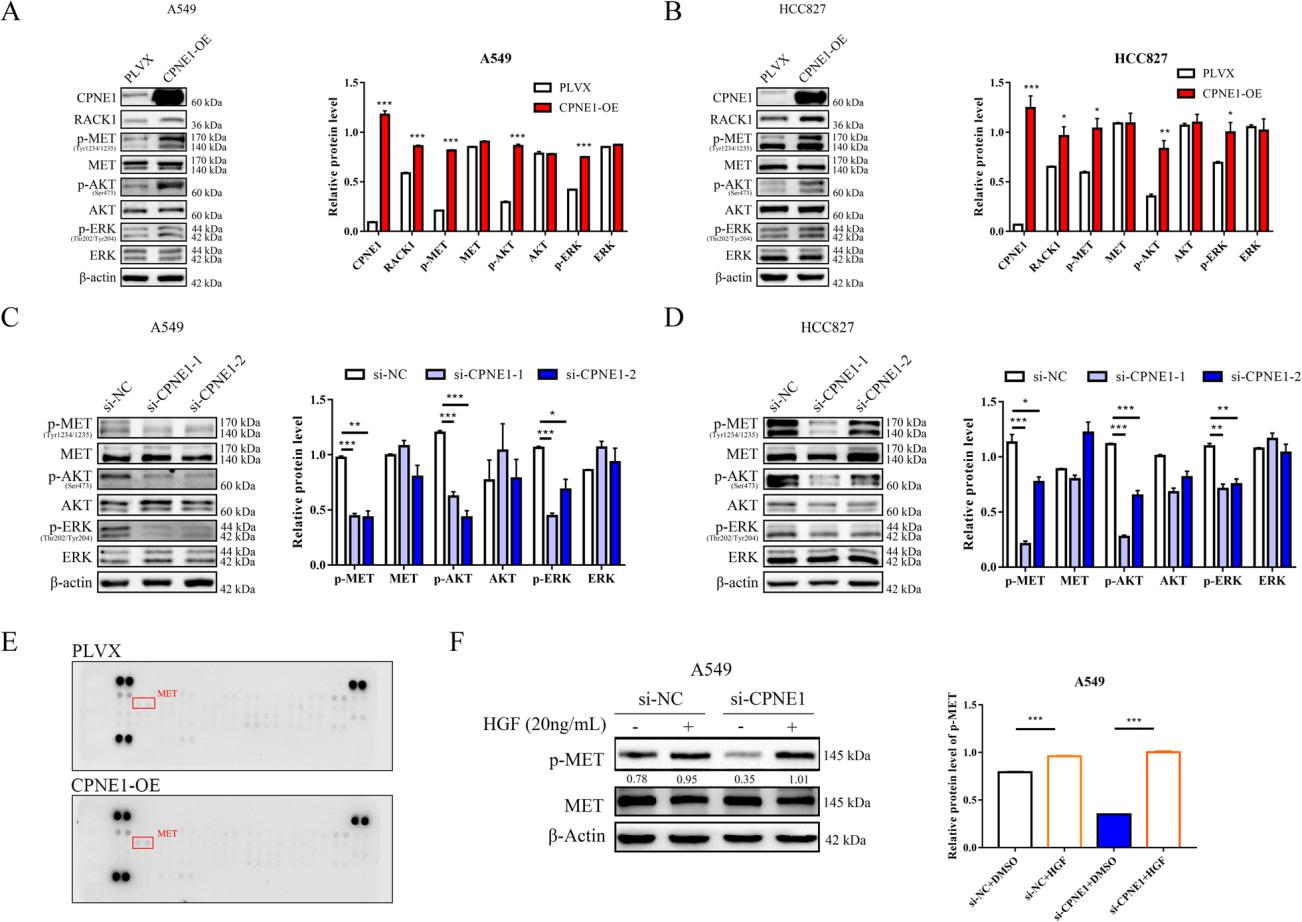
CPNE1 activated the MET signaling pathway in NSCLC. **A**, **B** Western blot analysis of RACK1, p-MET, MET, p-AKT, AKT, p-Erk, and Erk protein levels in CPNE1-overexpressing cells compared to control cells, with quantification on the right. **C**, **D** Western blot analysis of relevant protein levels in CPNE1 knockdown cells compared to control cells, with quantification on the right. **E** A human RTK phosphorylation array analysis demonstrated that the p-MET level is increased in CPNE1-overexpressing cells. **F** Western blot analysis of p-MET in CPNE1 knockdown cell lines treated with HGF (20 ng/mL) for 24 h. *P < 0.05; **P < 0.01; ***P < 0.001

Next, a human RTK phosphorylation array was performed in PLVX vector- and CPNE1-overexpressing cells to identify changes in potential downstream signaling pathways. The results showed that the phosphorylation of MET was upregulated after CPNE1 overexpression (Fig. 3E). Therefore, we determined the levels of p-MET, MET and other important signaling molecules (p-AKT, AKT, p-ERK, ERK) by western blot. The results showed that overexpression of CPNE1 significantly increased the signaling molecules in A549 and HCC827 cells (Fig. 3A, B), and knockdown of CPNE1 led to a significant decrease in A549 and HCC827 cells (Fig. 3C, D). Exogenous HGF treatment can stimulate MET signaling, while the HGF-induced increase in the level of p-MET was inhibited in CPNE1-knockdown cell lines (Fig. 3F). Consequently, these results supported the idea that CPNE1 promotes NSCLC progression by regulating MET signaling pathway in NSCLC. However, the underlying mechanism by which CPNE1 stimulated MET signaling remained unknown.

### CPNE1 interacts with RACK1 in NSCLC cells

**Fig. 4:**
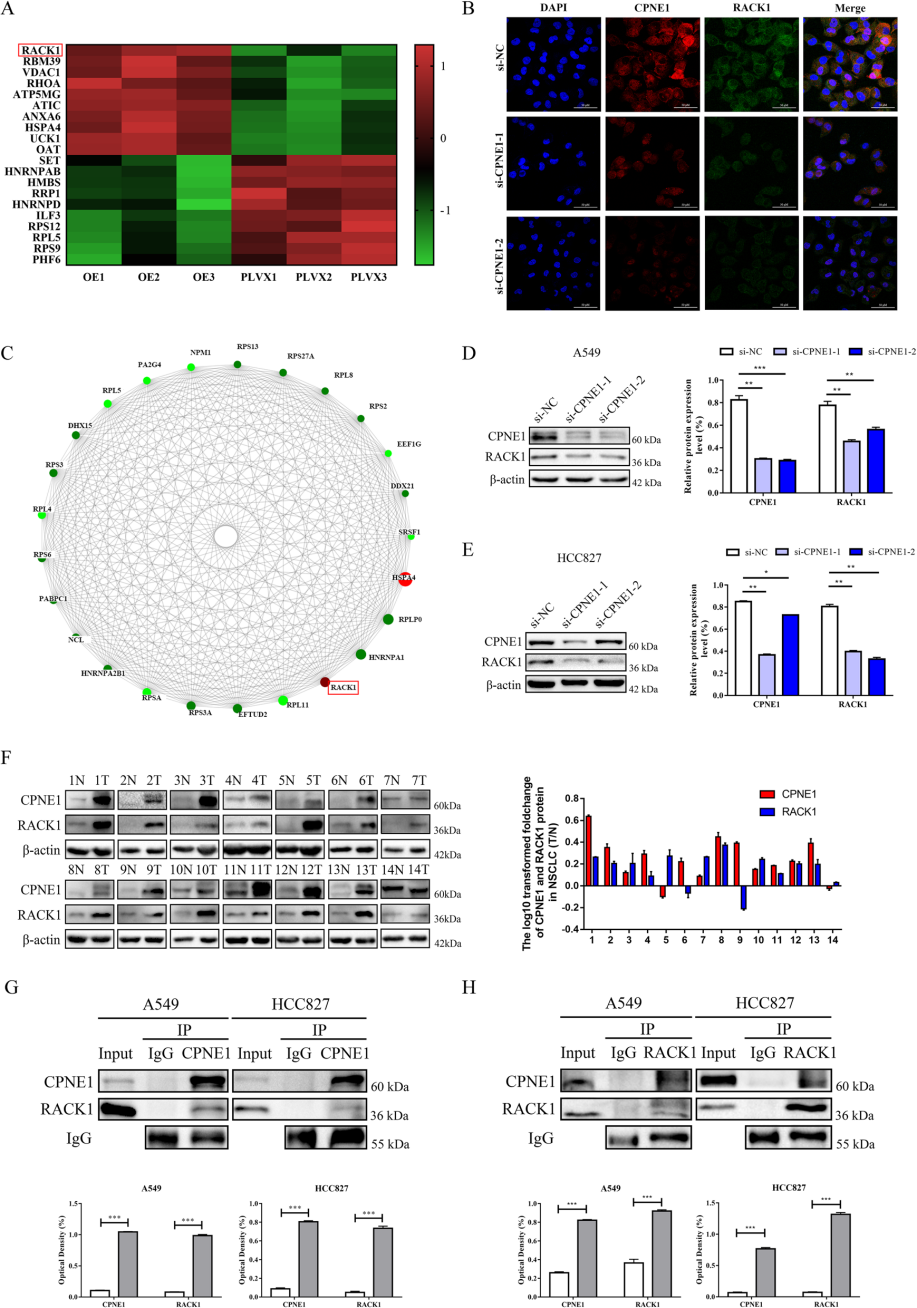
CPNE1 is positively related to RACK1 in NSCLC. **A** Differentially expressed proteins in PLVX- and CPNE1-overexpressing cells assessed by the mass spectrometry assay. **B** Immunofluorescence staining of CPNE1 and RACK1 in NSCLC cells. **C** The 25 most highly connected differentially expressed proteins in the protein–protein interaction analysis. **D**, **E** RACK1 protein levels were decreased in CPNE1-knockdown A549 and HCC827 cells. **F** Western blot analysis of CPNE1 and RACK1 protein levels in 14 paired NSCLC tissues and adjacent tissues. The right panel shows the relative quantification of CPNE1 and RACK1 protein levels. **G**, **H** CPNE1 and RACK1 interactions were detected in NSCLC cells by co-IP assays. *P < 0.05; **P < 0.01; ***P < 0.001

To further determine the underlying mechanism of CPNE1 and MET in NSCLC, we performed a mass spectrometry assay in PLVX and CPNE1-OE cells. The results showed that RACK1 was highly expressed in CPNE1-overexpressing cells (Fig. 4A), indicating that it is the most likely protein to interact with CPNE1 (Fig. 4C). Immunofluorescence staining determined that CPNE1 and RACK1 were colocalized in NSCLC cells (Fig. 4B). Western blot analysis confirmed that knockdown of CPNE1 reduced RACK1 expression (Fig. 4D, E). Moreover, we performed co-immunoprecipitation experiments to confirm the direct relationship between CPNE1 and RACK1. We found that CPNE1 specifically interacted with RACK1 and vice versa (Fig. 4G, H). Then, we further verified the relationship between CPNE1 and RACK1 in 14 paired NSCLC and adjacent lung tissue samples. Among these pairs, 10 pairs (71.4%) showed consistently upregulated CPNE1 and RACK1 expression in tissue samples (Fig. 4F). These results revealed a positive correlation between CPNE1 and RACK1 in NSCLC tissue as well as a direct interaction between the two in NSCLC cell lines which also indicated us it might be possible that CPNE1 activates MET through RACK1.

### Knockdown of RACK1 inhibits NSCLC cell proliferation, migration and invasion

**Fig. 5:**
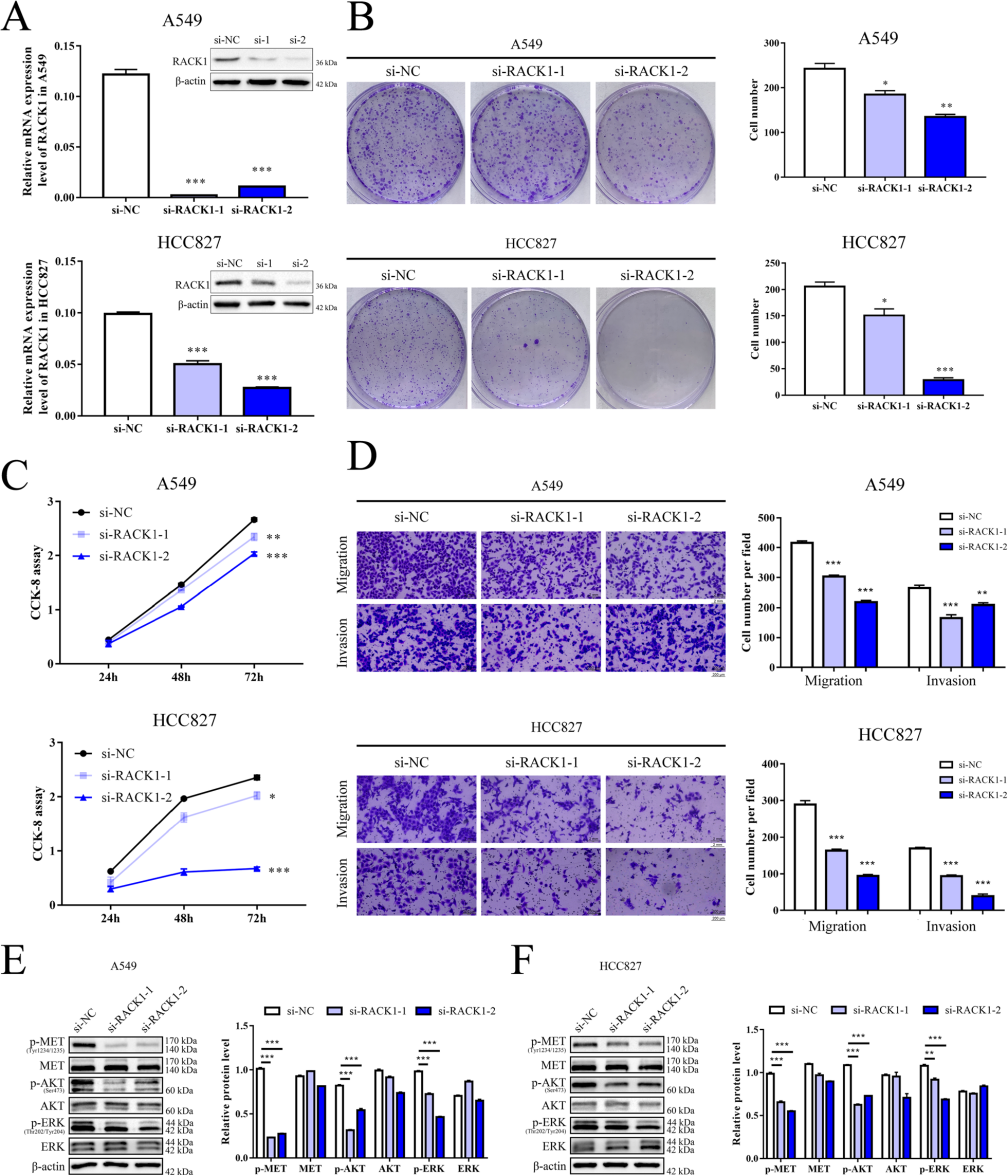
Knockdown of RACK1 inhibited the proliferation and motility of A549 and HCC827 cells. **A** The mRNA and protein level of RACK1 was decreased in RACK1 knockdown cells. **B** Clonogenic assays were performed to assess cell proliferation, and quantified on the right. **C** CCK-8 assays were also performed to analyse cell proliferation. **D** Transwell assays were performed to analyse the cell motility abilities. Data represent the mean (± SD) of three independent experiments, and unpaired *t* test was used to verify the statistical significance. **E**, **F** Western blot analysis of relevant protein levels in RACK1 knockdown cells compared to control cells, with quantification on the right. *P < 0.05; **P < 0.01; ***P < 0.001

RACK1 is known to play an important but dual role in tumour progression. It is downregulated and acts as a tumour suppressor in gastric cancer [27], whereas it is a recognized oncogene in other types of cancer [12, 13]. Thus, we knocked down RACK1 expression by siRNA in A549 and HCC827 cells to determine its function in NSCLC (Fig. 5A). CCK-8 assays and clonogenic assays showed that downregulation of RACK1 suppressed NSCLC cell proliferation in vitro (Fig. 5B, C). Transwell assays of A549 and HCC827 cells further indicated that loss of RACK1 reduced the migration and invasion abilities of NSCLC cells (Fig. 5D). Moreover, knockdown of RACK1 inhibited the level of p-MET and other important downstream signaling molecules (p-AKT and p-ERK) (Fig. 5E, F). Taken together, these observations indicated that aberrant regulation of RACK1 expression can affect tumour progression through promoting MET signaling in NSCLC.

### CPNE1 activates the MET signaling pathway through RACK1

**Fig. 6:**
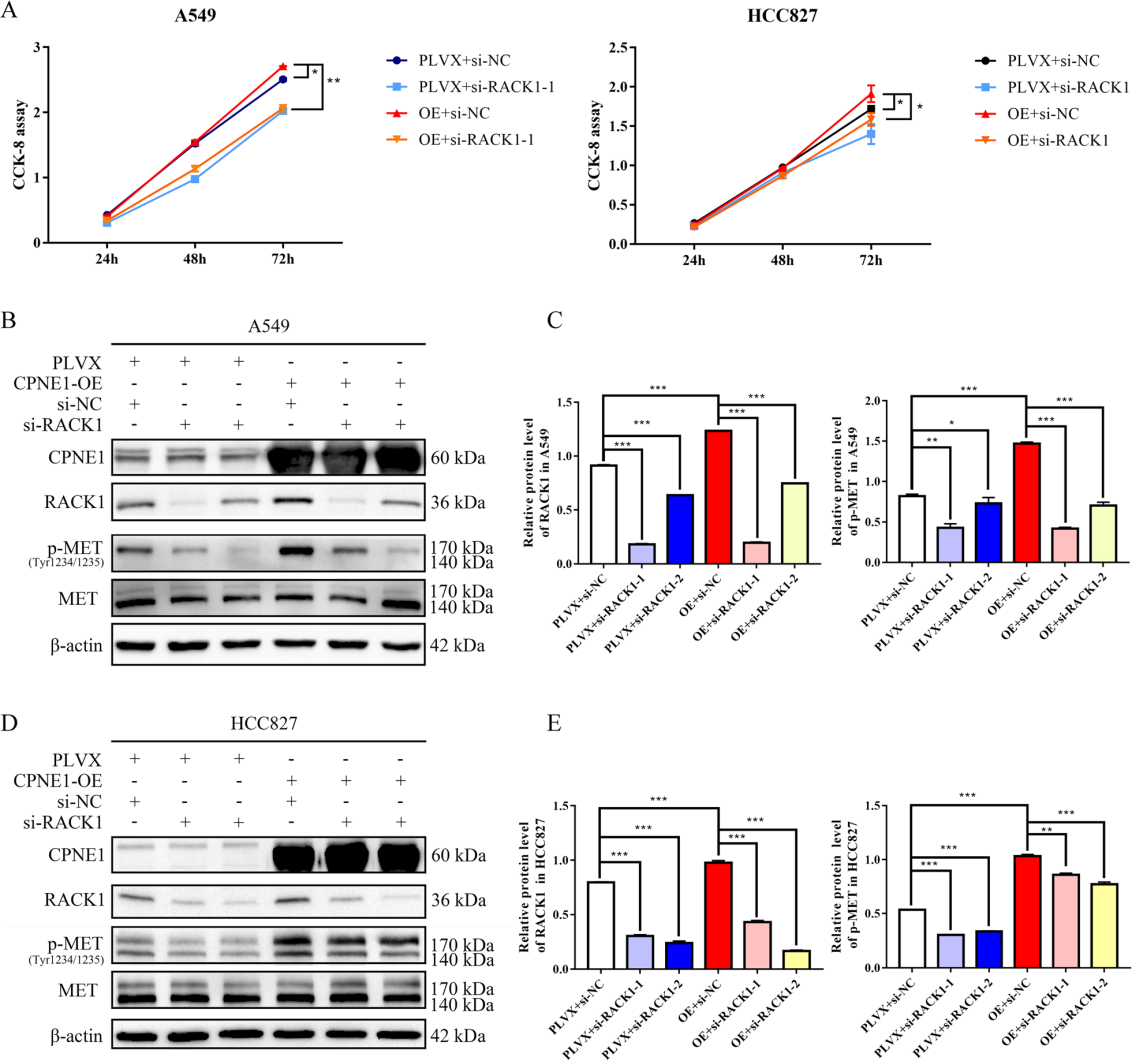
CPNE1 promotes cell proliferation by activating MET via interaction with RACK1. **A** Knockdown of RACK1 inhibits CPNE1-induced cell proliferation. **B**, **C** Knockdown of RACK1 inhibits CPNE1-induced activation of the MET signaling pathway in A549 cells. **D**, **E** Knockdown of RACK1 inhibits CPNE1-induced activation of the MET signaling pathway in HCC827 cells. *P < 0.05; **P < 0.01; ***P < 0.001

We also performed rescue experiments to establish the regulatory axis of CPNE1/RACK1/MET in NSCLC. First, the western blot results showed that inhibition of RACK1 decreased the p-MET, p-AKT and p-ERK levels in parental A549 and HCC827 cells, indicating that RACK1 can also activate the MET signaling pathway (Fig. 5E, F). Then, we transfected RACK1 siRNAs into CPNE1-overexpressing and control cells and found that knocking down RACK1 rescued the abnormally regulated RACK1 and p-MET levels (Fig. 6B–E). In addition, the CCK-8 assay demonstrated that reduced RACK1 expression can rescue the hyperactive cell proliferation induced by CPNE1 overexpression (Fig. 6A). Negative results of the HGF ELISA assay also suggested that CPNE1 activated MET signaling through RACK1 rather than HGF secretion (Additional file 5). These findings strongly suggest that CPNE1 plays an oncogenic role by interacting with RACK1 and then activating the MET signaling pathway in NSCLC.

### Inhibition of MET signaling blocks CPNE1-induced aberrant activation

**Fig. 7:**
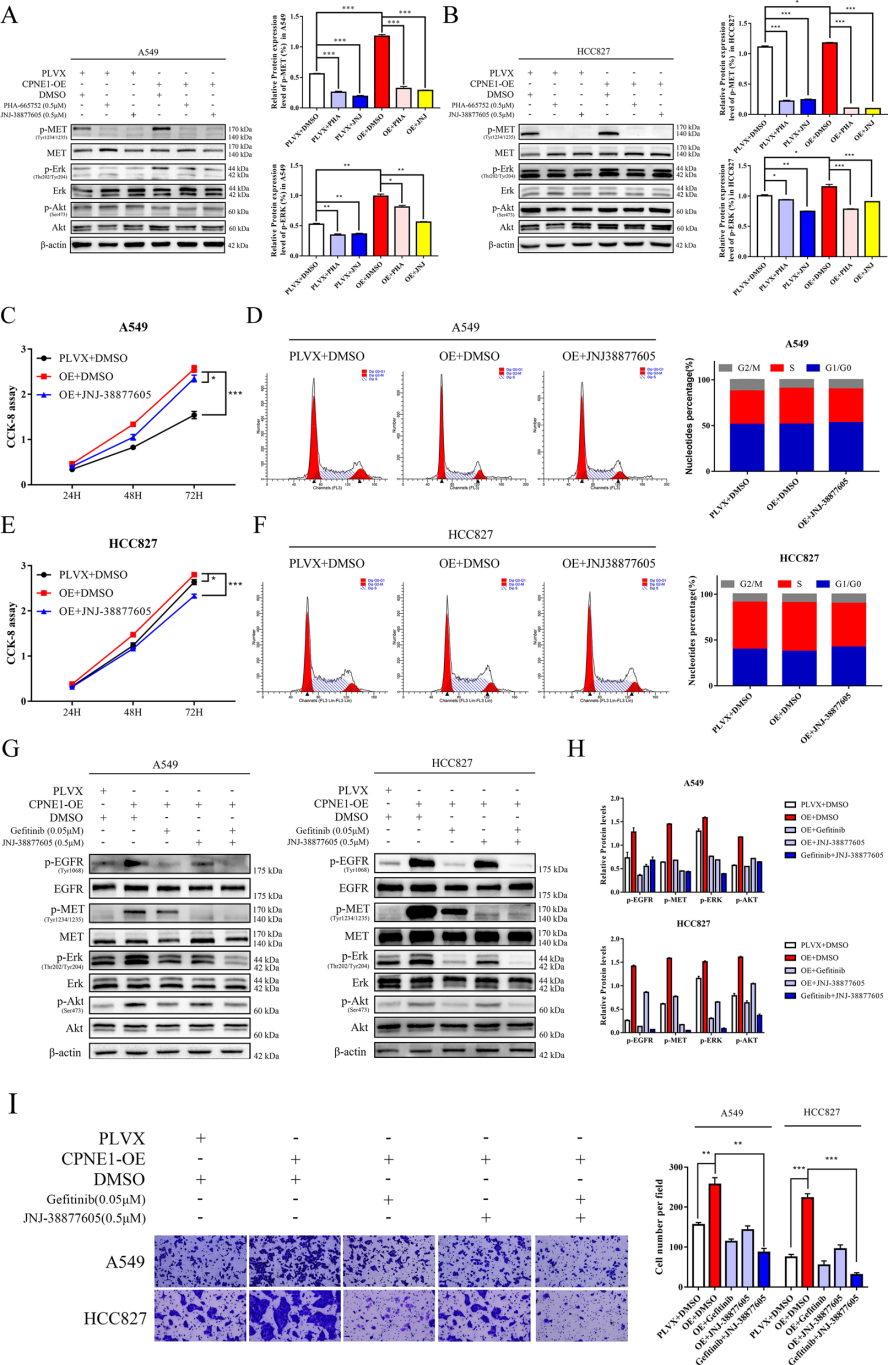
Inhibition of MET and EGFR blocks CPNE1-induced aberrant activation. **A** Inhibition of MET inhibits CPNE1-induced activation of the MET signaling pathway in A549 cells. **B** Inhibition of MET inhibits CPNE1-induced activation of the MET signaling pathway in HCC827 cells. **C**, **E** Inhibition of MET inhibits CPNE1-induced aberrant activation of cell proliferation. **D**, **F** Flow cytometric analysis showed MET inhibitors changed the percentage of cells in each cell cycle phase in CPNE1-overexpressing cell lines, as quantified on the right. **G**, **H** Dual blockade of MET and EGFR resulted in enhanced inhibition of downstream AKT and ERK pathways. **I** Dual blockade of MET and EGFR enhanced the inhibition of cell migration ability. *P < 0.05; **P < 0.01; ***P < 0.001

Then, we extend our study to the application of MET inhibitors and novel treatment strategies. JNJ-38877605 and PHA-665752 are two selective small-molecule MET inhibitors [28–30]. Both CPNE1-overexpressing and control cells were treated with 0.5 μM JNJ-38877605 and PHA-665752, respectively, for 24 h, and then the cell lysate was collected for WB analyses. The results showed that the phosphorylation of MET and ERK was significantly reduced by MET-specific inhibitors (Fig. 7A, B). The CCK-8 assay demonstrated that MET inhibitor can rescue the hyperactive cell proliferation induced by CPNE1 overexpression (Fig. 7C, E) and lessen the high proportion of cells in the S phase (Fig. 7D, F). However, we did not observe any evident changes in the p-AKT level. This brought us to think if combined treatment with other drugs could enhance the inhibitory effects. Our previous work revealed that CPNE1 can mediate the EGFR pathway [4] and that MET acts in cooperation with EGFR or is activated as a compensatory pathway in the presence of EGFR blockade [31]; hence, we intended to examine the effectiveness of combining the MET inhibitor JNJ-38877605 with the EGFR inhibitor gefitinib. Combined targeting of MET and EGFR resulted in enhanced inhibition of downstream AKT and ERK pathways as well as cell migration (Fig. 7G–I). These results both suggest that MET inhibitors can prevent CPNE1-induced abnormal molecular signaling and shed light on new joint therapeutic strategies.

### Dual inhibition of MET and EGFR suppressed CPNE1-induced tumour growth

**Fig. 8:**
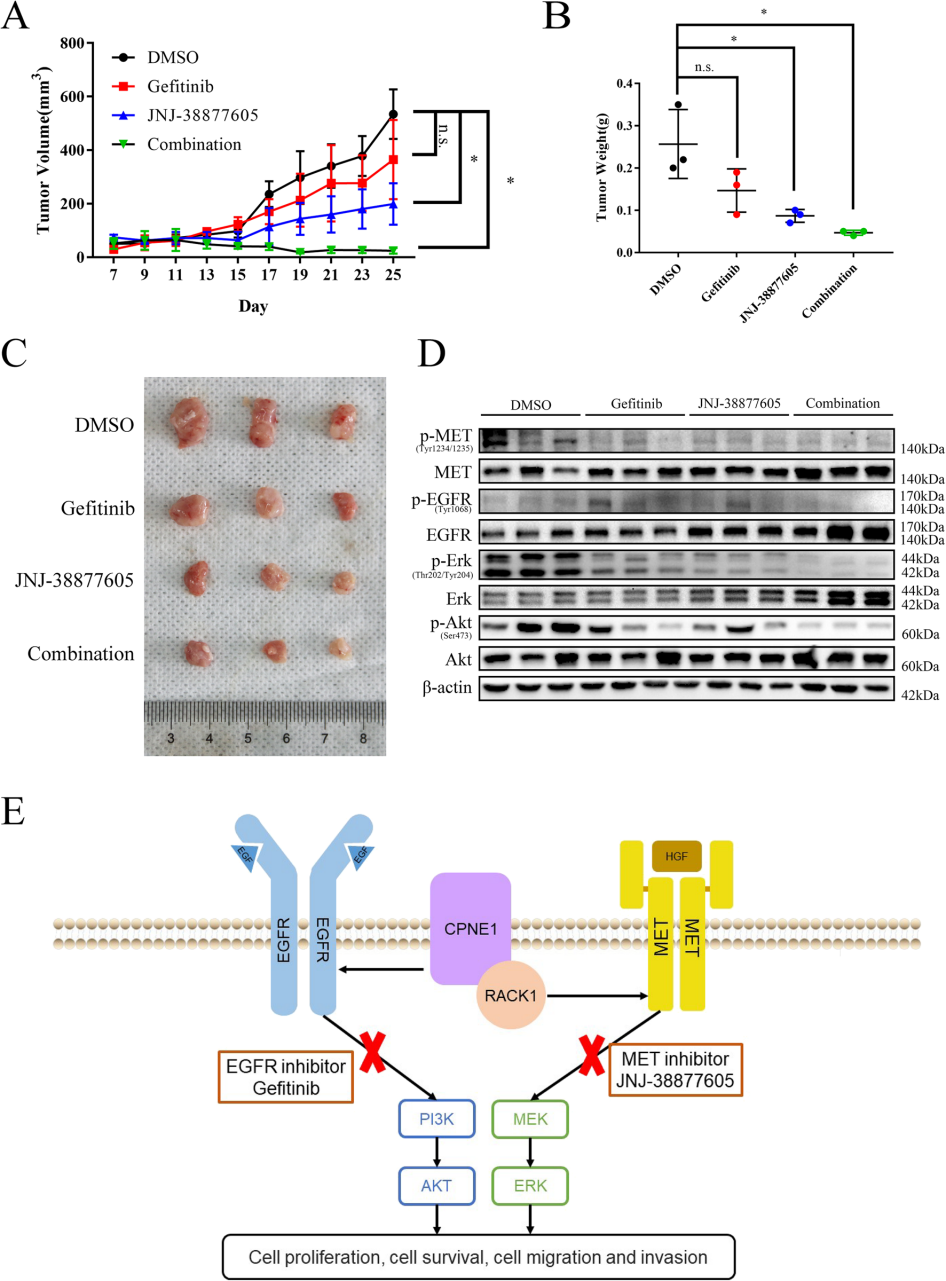
Dual blockade of MET and EGFR inhibited NSCLC growth in vivo*.* Athymic nude mice bearing A549 CPNE1-overexpressing tumours were treated with gefitinib, JNJ-38877605 or both for 2 weeks. **A**, **B** The tumours were measured and weighed. Data represent the mean (± SD), and unpaired *t* test was used to verify the statistical significance at the last point. **C** Xenograft tumours were dissected and photographed. **D** Western blotting was used to analyse the protein levels of p-EGFR, p-MET, p-AKT and p-ERK in tumour tissues of different treatment groups. **E** A diagram showing that CPNE1 mediates NSCLC progression by interacting with RACK1 and activating MET signaling. *P < 0.05; **P < 0.01; ***P < 0.001

To further determine that combined treatment with a MET inhibitor and gefitinib is superior to either monotherapy, we transplanted A549 cells overexpressing CPNE1 into subcutaneous sites in the flanks of immunocompromised mice. Mice with established tumours were then randomly divided into 4 groups and treated with different drugs. JNJ-38877605 was chosen for further in vivo experiments because it can be administered orally and PHA-665752 caused tissue damage at the injection site in previous research [28]. Tumour volumes were measured every other day (Fig. 8A). Tumours were captured and weighed after the mice were sacrificed (Fig. 8B, C). Although single treatment with gefitinib alone showed no significant effects, possibly because A549 cells were EGFR wild-type cells, combining JNJ-38877605 with gefitinib suppressed tumour growth significantly more than JNJ-38877605 alone. We further performed western blotting to verify that the inhibition of AKT and ERK phosphorylation was significantly stronger in the combination group (Fig. 8D). These results suggest that dual blockade of MET and EGFR may be a promising clinical therapeutic strategy for patients with high CPNE1 levels and extend the clinical usage of MET and EGFR inhibitors.

## Discussion

As long as the mortality of lung cancer remains high and its 5-year survival remains poor [1, 3], there is still an urgent need to further explore the underlying molecular mechanism of NSCLC development and progression. In the present study, we first demonstrated that CPNE1 interacts with RACK1 to further activate the MET signaling pathway and proposed a novel combination treatment strategy with MET and EGFR inhibitors in NSCLC (Fig. 8E).

CPNE1 is a soluble membrane-binding protein that exhibits a broad tissue distribution. It contains two C2 domains and an A domain through which it can bind with different intracellular proteins [8, 32]. CPNE1 is significantly upregulated and plays an oncogenic role in a variety of cancers, including breast cancer, colorectal cancer and prostate cancer [6]. Only 3 articles have reported the study of CPNE1 in lung cancer, and all were written by our research group. Our previous work demonstrated that CPNE1 can be upregulated by microRNAs and promotes NSCLC progression [4, 5, 33]. However, we have not elucidated the exact underlying mechanism by which CPNE1 plays its oncogenic role. Here, we first verified that CPNE1 can promote cell proliferation, migration and invasion in NSCLC cells, consistent with our previous findings. Protein mass spectrometry was then performed to investigate the potential protein–protein interactions and downstream signaling pathways activated by CPNE1. Interestingly, our experimental results confirmed that the overexpression of CPNE1 significantly increased the levels of RACK1 and p-MET, which provided new insights into the molecular mechanism of CPNE1. Thus, we continued our study focusing on the CPNE1/RACK1/MET axis.

RACK1 is a scaffolding protein known to regulate multiple processes via different pathways involved in tumorigenesis. Although many studies have reported the crucial role of RACK1 in tumorigenesis, its biological function varies in different types of cancers. In gastric and colorectal cancer, RACK1 is reported to be a tumour suppresser [27, 34]. On the other hand, RACK1 is recognized as an oncogene and promotes cell proliferation in hepatocellular cancer. It also enhances the invasion and metastasis of breast cancer cells. However, the context-dependent role of RACK1 in lung cancer remains controversial [16, 35]. In the current study, we found that loss of RACK1 inhibits the proliferation, migration and invasion abilities of NSCLC cells, consistent with most currently published papers [36]. Our research further validated the oncogenic role of RACK1 in NSCLC.

Subsequently, we performed immunofluorescence staining and co-IP assays to confirm the interaction between CPNE1 and RACK1. Western blot analysis also showed that RACK1 expression was positively related to CPNE1 expression in both cell lines and tissue samples. Furthermore, we found that knockdown of RACK1 reduced cell proliferation and MET signaling in CPNE1-overexpressing cells, indicating that the tumorigenic function of CPNE1 depends at least partially on RACK1. Interestingly, more literature research revealed that C2 regulatory regions, which are known to be conserved in C2 domains of CPNE1, were the first protein domains identified capable of interacting with RACK1 [37]. Therefore, it might be possible that RACK1, which is able to direct protein kinase Cs (PKCs) to specific membrane locations [38], might also directly interact with CPNE1. Taken together, we established the hypothesis that CPNE1 promotes cell proliferation and motility in NSCLC by interacting with RACK1 via MET signaling activation.

While the MET pathway facilitates embryogenesis and tissue regeneration under physiological conditions [18], aberrantly activated MET signaling is known to promote tumorigenesis in various cancers, including NSCLC [39, 40]. It mediates several biological processes in cancer, such as cell proliferation, metastasis and drug resistance, via regulation of downstream signaling pathways, such as MEK/ERK and PI3K/AKT [17, 20, 41]. A variety of gene alterations contribute to aberrant MET signaling. MET exon 14 skipping mutation (METex14), an oncogenic driver in approximately 3–4% of NSCLCs [42, 43], and MET amplification, found in 20% of EGFR-TKI-resistant NSCLC patients [44, 45], are often detected and frequently studied in NSCLC. Thus, it is of interest to investigate a new cure for the MET signaling pathway that might inhibit tumorigenesis and reverse drug resistance at the same time. In this study, a human RTK phosphorylation array suggested that CPNE1 might achieve its biological functions via MET signaling. Further experiments verified that the phosphorylation level of MET varies according to the alteration of CPNE1 expression. Loss of RACK1 suppressed the abnormal p-MET level induced by CPNE1 overexpression. However, the inhibition of the downstream AKT pathway was not ideal when CPNE1-overexpressing cells were treated with a specific MET inhibitor. Our previous findings demonstrated that CPNE1 might also mediate EGFR signaling, which prompted us to consider the possibility of combination treatment with MET and EGFR inhibitors. Compared with either single-agent treatment, combined targeting of MET and EGFR resulted in enhanced reductions in tumour volumes and weights, accompanied by decreased activation of downstream signaling pathways in vivo*,* and the results were consistent with those of the in vitro experiments. These results suggest that dual blockade of MET and EGFR may be a promising clinical therapeutic strategy for treating NSCLC.

There were still limitations to this study in that the exact mechanism by which CPNE1, RACK1 and MET interact with each other needs further study. The interaction between CPNE1 and RACK1, which might be dependent on a specific C2 regulatory domain, was not fully investigated. Hopefully, we will solve these problems in our follow-up studies.

## Conclusions

In summary, we identified an oncogene, CPNE1, that promotes cell proliferation, migration and invasion in NSCLC. Moreover, our study demonstrated for the first time that CPNE1 promotes tumorigenesis via the MET signaling pathway by interacting with RACK1. Collectively, the findings of our study offer mechanistic insights into the oncogenic roles of CPNE1 and RACK1 in NSCLC and suggest that dual blockade of MET and EGFR may be a promising clinical therapeutic strategy for NSCLC.

## Supplementary Information

**Additional file 1: Table S1.** Sequences of Primers for Real-time Polymerase Chain Reaction.

**Additional file 2: Table S2.** Sequences of siRNAs.

**Additional file 3: Table S3.** The list of specific kinase targets screened in the human RTK phosphorylation array.

**Additional file 4: Fig S1.** High expression levels of CPNE1 in NSCLC tissues and cell lines. (A) Statistics on CPNE1 mRNA expression in lung adenocarcinoma, squamous cell carcinoma and normal lung tissue from GEPIA database (http://gepia.cancer-pku.cn/). (B)The relation of CPNE1 and overall survival in 478 lung adenocarcinoma patients in the GEPIA2 database. (C, D) The expression levels of CPNE1 in 6 NSCLC cell lines and a bronchial epithelial cell line were examined by qRT-PCR and western blot analysis.

**Additional file 5.** ELISA was used to detect expression of HGF in A549 and HCC827 cells.

## Data Availability

The datasets used and/or analysed during the current study are available from the corresponding author on reasonable request.

## Abbreviations

NSCLC: Non-small cell lung cancer
CPNE1: Copine 1
RACK1: Receptor for activated C kinase 1
EGFR: Epidermal growth factor receptor
WD-repeat: Tryptophan-aspartate repeat
Gβ: β Subunit of G-proteins
RTK: Receptor tyrosine kinase
HGF: Hepatocyte growth factor
EGFR-TKIs: Epidermal growth factor receptor tyrosine kinase inhibitors
qRT-PCR: Real-time quantitative reverse transcription PCR
PKCs: Protein kinase Cs
METex14: MET exon 14 skipping mutation
