# Supplementary material for: CPNE1 promotes non-small cell lung cancer progression by interacting with RACK1 via the MET signaling pathway
Source: Cell Commun Signal. 2022 Jan 31;20:16. doi: 10.1186/s12964-021-00818-8 (PMC8802424; doi:10.1186/s12964-021-00818-8)
Supplement: Supplementary file 2 — Additional file 1: Table S1. Sequences of Primers for Real-time Polymerase Chain Reaction. [file 12964_2021_818_MOESM2_ESM.docx]

Additional file 2: Table S1. Sequences of Primers for Real-time Polymerase Chain Reaction.

| Gene | Forward | Reverse |
| --- | --- | --- |
| CPNE1 | 5′-ACCCACTCTGCGTCCTT-3′ | 5′-TGGCGTCTTGTTGTCTATG-3′ |
| RACK1 | 5′-TGAGTGTGGCCTTCTCCTCT-3′ | 5′-AAAGGTGTTTGCCTTCGTTG-3′ |
| β-actin | 5′-CCTGGCACCCAGCACAAT-3′ | 5′-GGGCCGGACTCGTCATAC-3′ |
