# Supplementary material for: CPNE1 promotes non-small cell lung cancer progression by interacting with RACK1 via the MET signaling pathway
Source: Cell Commun Signal. 2022 Jan 31;20:16. doi: 10.1186/s12964-021-00818-8 (PMC8802424; doi:10.1186/s12964-021-00818-8)
Supplement: Supplementary file 3 — Additional file 2: Table S2. Sequences of siRNAs. [file 12964_2021_818_MOESM3_ESM.docx]

Additional file 3: Table S2. Sequences of siRNAs.

| siRNA | Sense | Anti-sense |
| --- | --- | --- |
| Si-NC | 5′-UUCUCCGAACGUGUCACGUTT-3′ | 5′-ACGUGACACGUUCGGAGAATT-3′ |
| Si-CPNE1-1 | 5′-GGACUUCACUGGCUCCAAUTT-3′ | 5′-AUUGGAGCCAGUGAAGUCCTT-3′ |
| Si-CPNE1-2 | 5′-GCAGGUCUCGCAUGAAUUUTT-3′ | 5′-AAAUUCAUGCGAGACCUGCTT-3′ |
| Si-RACK1-1 | 5′-GCAAACACCUUUACACGCTT-3′ | 5′-GCGUGUAAAGGUGUUUGCTT-3′ |
| Si-RACK1-2 | 5′-CAGAUUGUCUCUGGAUCUCGA-3′ | 5′-UCGAGAUCCAGAGACAAUCUG-3′ |
