# Supplementary material for: CPNE1 promotes non-small cell lung cancer progression by interacting with RACK1 via the MET signaling pathway
Source: Cell Commun Signal. 2022 Jan 31;20:16. doi: 10.1186/s12964-021-00818-8 (PMC8802424; doi:10.1186/s12964-021-00818-8)
Supplement: Supplementary file 4 — Additional file 3: Table S3. The list of specific kinase targets screened in the human RTK phosphorylation array. [file 12964_2021_818_MOESM4_ESM.docx]

Additional file 4: Table S3. The list of specific kinase targets screened in the human RTK phosphorylation array.


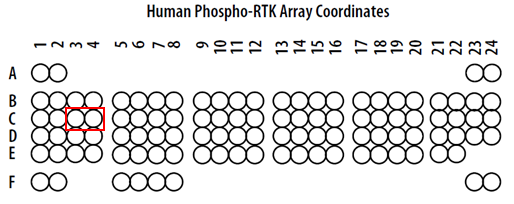


|  | 1 | 2 | 3 | 4 | 5 | 6 | 7 | 8 | 9 | 10 | 11 | 12 | 13 | 14 | 15 | 16 | 17 | 18 | 19 | 20 | 21 | 22 | 23 | 24 |
| --- | --- | --- | --- | --- | --- | --- | --- | --- | --- | --- | --- | --- | --- | --- | --- | --- | --- | --- | --- | --- | --- | --- | --- | --- |
| A | Reference Spot | Reference Spot |  |  |  |  |  |  |  |  |  |  |  |  |  |  |  |  |  |  |  |  | Reference Spot | Reference Spot |
| B | EGFR | EGFR | ErbB2 | ErbB2 | ErbB3 | ErbB3 | ErbB4 | ErbB4 | FGFR1 | FGFR1 | FGFR2-α | FGFR2-α | FGFR3 | FGFR3 | FGFR4 | FGFR4 | Insulin R | Insulin R | IGF-IR | IGF-IR | Axl | Axl | Dtk | Dtk |
| C | Mer | Mer | MET | MET | MSPR | MSPR | PDGFR-α | PDGFR-α | PDGFR-β | PDGFR-β | SCFR | SCFR | Flt-3 | Flt-3 | M-CSFR | M-CSFR | c-Ret | c-Ret | ROR1 | ROR1 | ROR2 | ROR2 | Tie-1 | Tie-1 |
| D | Tie-2 | Tie-2 | TrkA | TrkA | TrkB | TrkB | TrkC | TrkC | VEGFR1 | VEGFR1 | VEGFR2 | VEGFR2 | VEGFR3 | VEGFR3 | MuSK | MuSK | EphA1 | EphA1 | EphA2 | EphA2 | EphA3 | EphA3 | EphA4 | EphA4 |
| E | EphA6 | EphA6 | EphA7 | EphA7 | EphB1 | EphB1 | EphB2 | EphB2 | EphB4 | EphB4 | EphB6 | EphB6 | ALK | ALK | DDR1 | DDR1 | DDR2 | DDR2 | EphA5 | EphA5 | EphA10 | EphA10 |  |  |
| F | Reference Spot | Reference Spot |  |  | EphB3 | EphB3 | RYK | RYK |  |  |  |  |  |  |  |  |  |  |  |  |  |  | Negative Control | Negative Control |
