# Supplementary figures and images for: CPNE1 promotes non-small cell lung cancer progression by interacting with RACK1 via the MET signaling pathway
Source: Cell Commun Signal. 2022 Jan 31;20:16. doi: 10.1186/s12964-021-00818-8 (PMC8802424; doi:10.1186/s12964-021-00818-8)

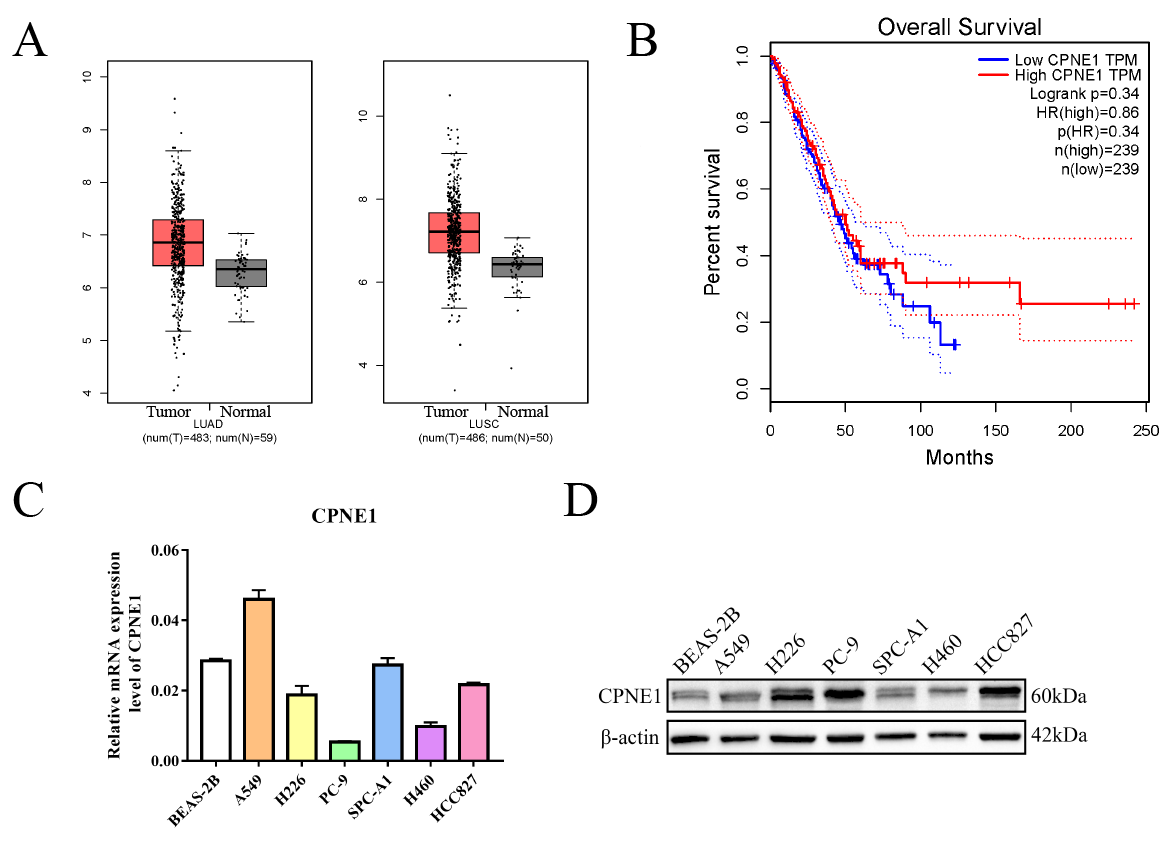

Supplement: Supplementary file 5 — Additional file 4: Fig S1. High expression levels of CPNE1 in NSCLC tissues and cell lines. (A) Statistics on CPNE1 mRNA expression in lung adenocarcinoma, squamous cell carcinoma and normal lung tissue from GEPIA database (http://gepia.cancer-pku.cn/). (B)The relation of CPNE1 and overall survival in 478 lung adenocarcinoma patients in the GEPIA2 database. (C, D) The expression levels of CPNE1 in 6 NSCLC cell lines and a bronchial epithelial cell line were examined by qRT-PCR and western blot analysis. [file 12964_2021_818_MOESM5_ESM.tif]

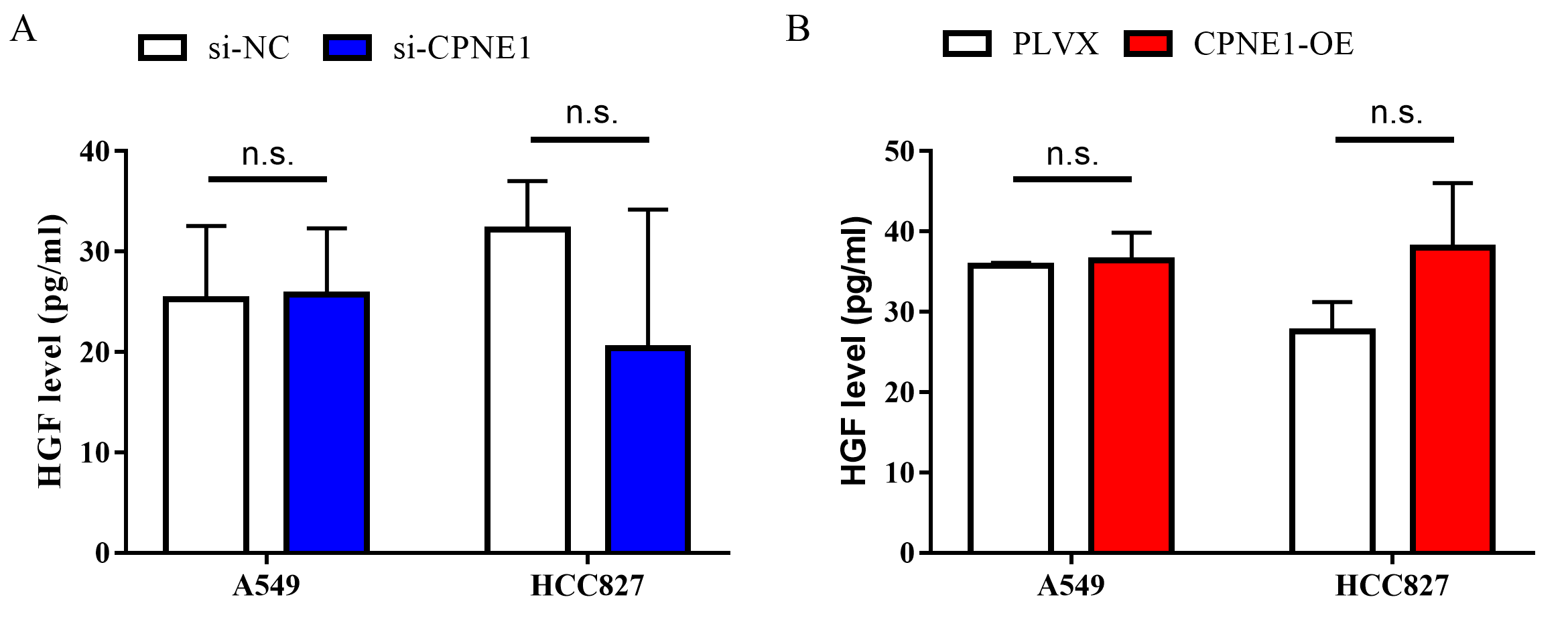

Supplement: Supplementary file 6 — Additional file 5. ELISA was used to detect expression of HGF in A549 and HCC827 cells. [file 12964_2021_818_MOESM6_ESM.tif]
